# The Role of Neutrophil NETosis in Organ Injury: Novel Inflammatory Cell Death Mechanisms

**DOI:** 10.1007/s10753-020-01294-x

**Published:** 2020-08-24

**Authors:** Zhen Cahilog, Hailin Zhao, Lingzhi Wu, Azeem Alam, Shiori Eguchi, Hao Weng, Daqing Ma

**Affiliations:** 1 Anaesthetics, Pain Medicine and Intensive Care, Department of Surgery and Cancer, Faculty of Medicine, Imperial College London, Chelsea and Westminster Hospital, 369 Fulham Road, London, SW10 9NH UK; 2 Department of Anesthesiology, Shanghai Fengxian District Central Hospital, Shanghai Jiao Tong University Affiliated Sixth People’s Hospital South Campus, Fengxian District, Shanghai, China

**Keywords:** NETosis, neutrophil, organ injury, cell death, inflammation

## Abstract

NETosis is a type of regulated cell death dependent on the formation of neutrophil extracellular traps (NET), where net-like structures of decondensed chromatin and proteases are produced by polymorphonuclear (PMN) granulocytes. These structures immobilise pathogens and restrict them with antimicrobial molecules, thus preventing their spread. Whilst NETs possess a fundamental anti-microbial function within the innate immune system under physiological circumstances, increasing evidence also indicates that NETosis occurs in the pathogenic process of other disease type, including but not limited to atherosclerosis, airway inflammation, Alzheimer’s and stroke. Here, we reviewed the role of NETosis in the development of organ injury, including injury to the brain, lung, heart, kidney, musculoskeletal system, gut and reproductive system, whilst therapeutic agents in blocking injuries induced by NETosis in its primitive stages were also discussed. This review provides novel insights into the involvement of NETosis in different organ injuries, and whilst potential therapeutic measures targeting NETosis remain a largely unexplored area, these warrant further investigation.

## BACKGROUND

Cell death is commonly segregated into necrosis and apoptosis; apoptosis being programmed cell death, for instance during development and physiological cellular turnover, whilst necrosis predominantly takes place in an unregulated manner [1]. NETosis, like necrosis, is a mode of cell death that involves the loss of membrane integrity. During NETosis, decondensation of chromatin is thought to be initiated by peptidyl arginine deiminase 4 (PAD4) [2]; its subsequent release together with granule contents is vital in the innate immune response to infection and inflammation. Recent studies suggest that NET formation is of central to pathogenesis of organ injury. This review will summarise the current understanding of the molecular mechanisms of NETosis and the therapeutic approaches under development targeting NET-induced organ injury.

## MOLECULAR MECHANISM OF NET FORMATION

Although NETosis is closely associated with NET formation, not all NET formation requires the process of cell death to take place beforehand. According to Nomenclature Committee on Cell Death, the term ‘NETosis’ should only be used in the context of cell death, and not just based on the presence of NET formation [3].

**Fig. 1:**
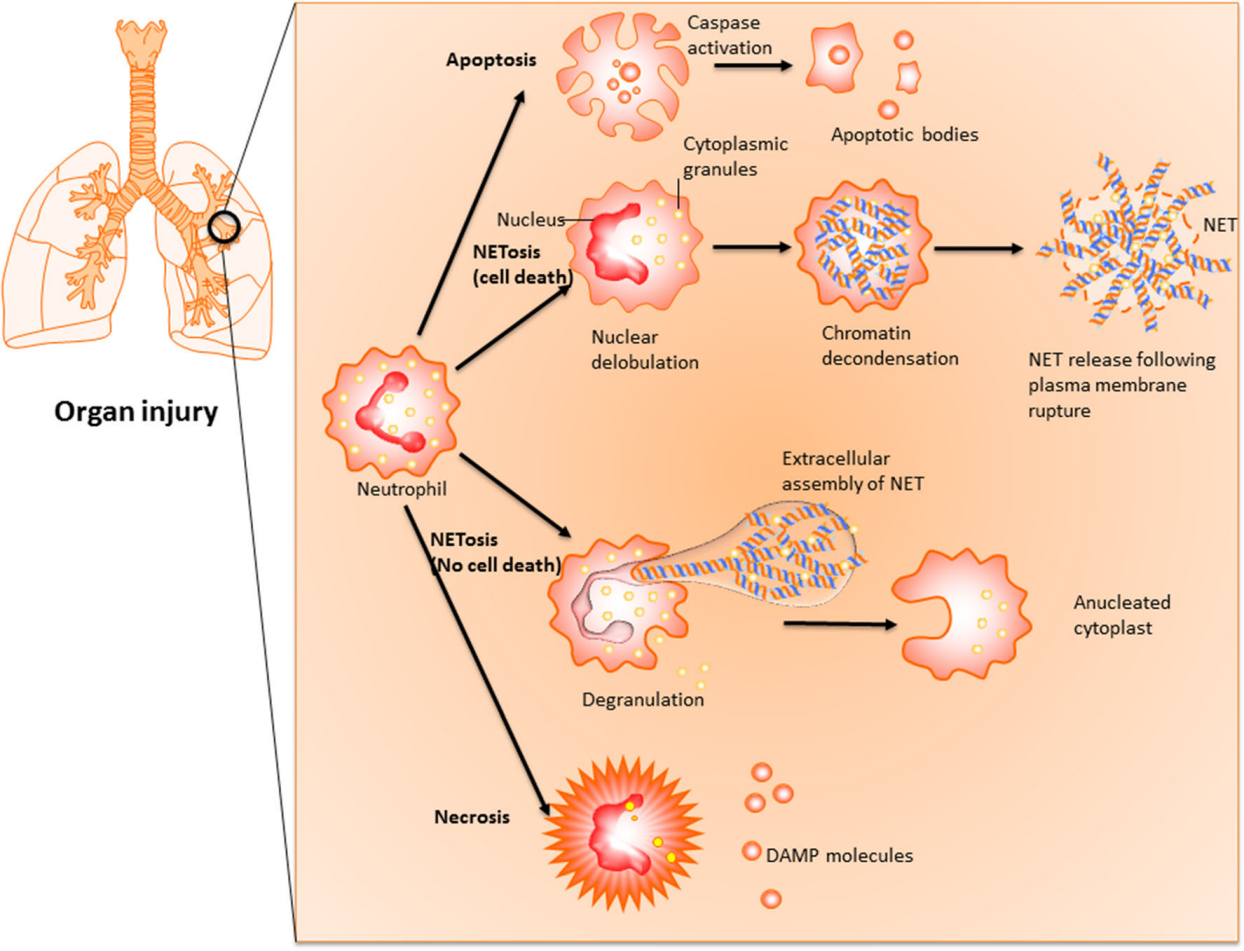
Type of cell death for neutrophil in organ injury. During solid organ injury, neutrophils could be prompted to undergo caspase-dependent apoptosis, which results in controlled dissolution of cell into apoptotic bodies containing cellular debris to prevent immune and inflammatory responses. Neutrophil extracellular traps (NETs) form *via* two pathways. The first is through lytic NETosis, a cell death pathway characterised by nuclear de-lobulation and disintegration of the nuclear envelope, which precedes loss of cellular polarisation, chromatin de-condensation and plasma membrane rupture. The second mechanism involves the non-lytic form of NETosis, which is not associated with cellular death but prompts expulsion of nuclear chromatin together with release of granule proteins through degranulation. These components can assemble in the extracellular space into NETs, leaving behind enucleated cytoplasts that continue to ingest microorganisms [4]. In addition, neutrophils could undergo unregulated necrosis that does not involve specific molecular pathways, with uncontrolled release of cellular debris acting as danger-associated molecular patterns (DAMPs) to trigger pro-inflammatory response.

Two main pathways of NET formation have been described and categorised according to their dependence on the activity of nicotinamide adenine dinucleotide phosphate (NADPH) oxidase pathway (Fig. 1) [5].

### NADPH Oxidase-Dependent NET Formation

The NADPH oxidase-dependent molecular pathway of NET formation begins with activation of neutrophil surface receptors by stimuli derived from pathogenic or non-pathogenic sources, such as cholesterol or urate, and ends with cellular lysis. These stimuli trigger calcium release from the endoplasmic reticulum (ER), resulting in the activation of protein kinase C (PKC) and the assembly of the NADPH oxidase complex generating reactive oxygen species (ROS) [6]. Following this, neutrophil elastase (NE), a protease stored in the cytoplasmic granules, migrates to the nucleus in a myeloperoxidase (MPO)-dependent manner and cleaves histones to initiate chromatin decondensation [7]. This is promoted by the citrullination of histone arginine residues by peptidylarginine deiminase IV (PAD4) [8]. Finally, mixing of the chromatin and granule proteins takes place as cellular membranes are broken down. Interestingly, there have been reports of mitochondrial DNA (mtDNA), instead of nuclear DNA, being the source of the DNA fibres in NETs, with observations of mtDNA being released from granulocytes in response to disease states such as trauma and systemic lupus erythematosus (SLE) [9, 10]. Moreover, it seems that histone citrullination is not always required for NET formation, as observed by Kenny and colleagues in their study of neutrophils activated by the PKC agonist phorbol 12-myristate 13-acetate (PMA); this highlights the diversity of pathways for NET formation following their induction [11].

Degradation of NETs can take place through several pathways, for example, by DNases [12], or endocytosed by macrophages [13].

Factors that influence NET formation include pH, CO_2_ and HCO_3_− levels, which modulate neutrophil activation. This explains why NETs form more readily in the periphery of an inflammatory site, where the pH is more alkaline [14]. This may influence treatment efficacy as NETs can seal off the affected area. An acidic environment has been hypothesised to reduce NADPH oxidase-dependent NET formation by reducing neutrophil glycolysis [15].

NADPH oxidase-dependent NET formation also requires neutrophils to be in the cell cycle, necessitating the activation of cyclin-dependent kinases (CDK) [16], phosphorylating the retinoblastoma protein.

### NADPH Oxidase-Independent NET Formation

This mechanism of NET formation is more relevant in the context of infection, as inducers of NETosis here are calcium ionophore A23187 and the potassium ionophore nigericin, which are products of *Streptomyces chartreusensis* and *Streptomyces hygroscopicus*, respectively [11]. How this pathway leads to NET release is poorly understood.

## NETosis AND INFLAMMATION

NETs under physiological conditions are central to pathogen clearance. When there is excessive formation or suboptimal, NETs are able to initiate further destructive signalling through interaction with other tissue constituents and the immune system. Moreover, the antimicrobial histones and peptides within the NET structure impose a direct cytotoxic effect on tissues [17]. To date, there have been numerous accounts of NETosis being present in diseases of major organs. Understanding of NETosis in pathophysiology may offer unique opportunities for clinical management.

## NETosis IN ORGAN INJURY

**Fig. 2:**
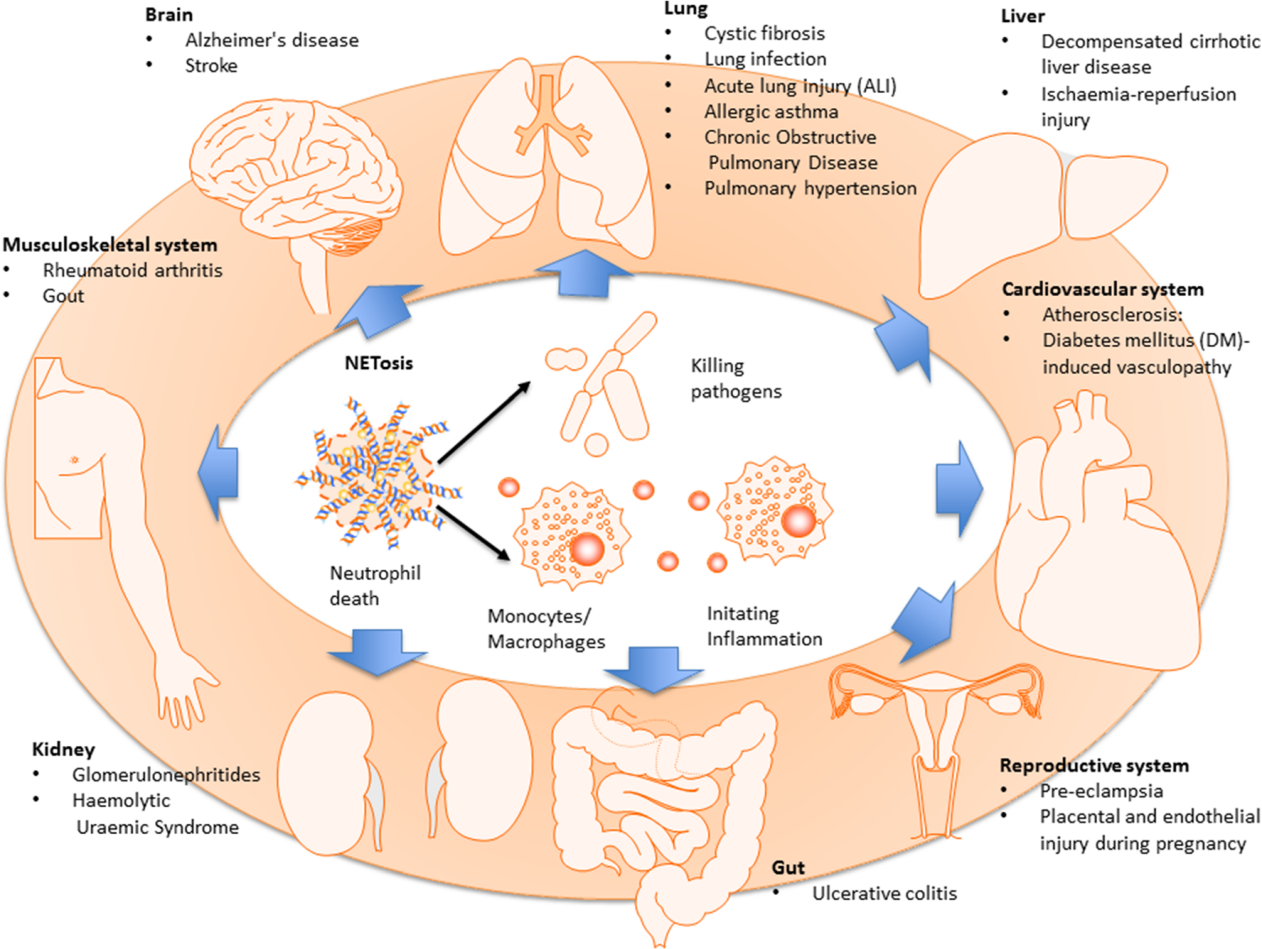
Involvement of NETosis in organ injury. Accumulating evidences now point to an important role of NETosis in infectious and non-infectious solid organ injury. Neutrophil invasion into brain parenchyma and release of neutrophil extracellular traps (NETs) have been established in the pathophysiology of Alzheimer’s disease to cause destruction to neural cells and blood-brain barrier. Abnormal NETosis activity and reactive oxygen species (ROS) response, a key element to NETosis initiation, were observed in stroke patients. The degree of neutrophil infiltration, NET formation/component (*e.g.* cell-free nucleosomes) and NETosis have been found to correlate with the severity of a range of lung diseases, including cystic fibrosis, acute lung injury (ALI)/acute respiratory distress syndrome (ARDS) and lung infection. NETosis was also shown to be involved in allergic asthma, chronic obstructive pulmonary disease and pulmonary hypertension, wherein degree of NET formation correlates with disease severity. During liver ischaemia-reperfusion, toll-like receptor-dependent NET release has been suggested to mediate liver inflammation and injury. Conversely, deficiency in NET release was reported in decompensated cirrhotic liver disease and could explain susceptibility to bacterial peritonitis infection in those patients. NET formation and NETosis have further been implicated in atherosclerosis and myocardial infarction, wherein NET was found in thrombi and infarct lesion and correlate with disease severity. In rheumatoid arthritis, enhanced NET release and NETosis are observed in synovial tissue, rheumatoid nodules and skin, whilst pro-inflammatory cytokines and autoantibodies further aggravate neutrophil infiltration and NETosis. Neutrophils could also be potently activated by monosodiumurate (MSU) crystals in gout joints and point to a potential role of NET/NETosis in gout pathogenesis. Moreover, neutrophil activation and NET deposition were also observed in colon mucosa of ulcerative colitis. Excessive neutrophil activation, NET formation and NETosis could also be responsible different pregnancy-related disorders, including pre-eclampsia wherein NET deposition and NETosis in the intervillous space may damage maternal endothelium and impair foetal oxygen exchange.

There is an expanding body of research describing NETosis in infectious and non-infectious organ injury (summarised in Fig. 2). Although it is valid that in these scenarios, nuclear DNA released during necrotic cell death can contribute to tissue injury and exacerbate the extent of organ damage, here we focus on the contribution by aberrant NET formation and the implication of understanding its underlying pathogenesis for therapeutic interventions.

### Brain

#### Alzheimer’s Disease

Alzheimer’s disease (AD) is a common disorder of neurodegeneration characterised by gradual loss of cognitive functions. In AD patients, neutrophils have been observed to invade the brain parenchyma and release NETs, causing destruction of neural cells and the blood-brain barrier [18].

#### Stroke

It is well known that the adaptive immune system is altered after a stroke, predisposing patients to infections [19–21]. Interestingly, NETosis has also been described as significantly impaired up until on day 5 in those with an ischaemic stroke [22]. Though NETosis in haemorrhagic stroke patients has yet to be documented, it has been noted that the generation of ROS, a key requirement for chromatin decondensation, is suppressed in these patients for up to 20 days [23].

### Lung

#### Cystic Fibrosis

It has been well established that chronic infections in cystic fibrosis (CF) patients are due to the highly viscous mucus production harbouring microbial growth. Although impaired clearance of mucus has been principally named responsible, there is increasing evidence that the high viscosity is also due vast amounts of free DNA, found in sputum samples [24], which was in concordance with the high concentration of neutrophil and NETs found in CF lungs. The source was believed to be from necrotic neutrophils [25] but is now considered to be secondary to NETosis [26]. Additionally, NET production was found to be promoted by bacterial infection in CF airways and was defective in clearance of the bacteria *Pseudomonas aeruginosa.* NETs are also named as a facilitative factor for biofilm formation. There is evidence that surfactant protein D (SP-D), responsible for opsonising pathogens and dying cells for clearance by alveolar macrophages [27], is essential for NET clearance, through binding directly to the chromatin within the NETs. SP-D levels are decreased in CF patients, and the level of decrease is proportional to the degree of inflammation, through accumulation of NETs [28].

#### Lung Infection

Neutrophils migrated into the affected site and initiate the cascade of antimicrobial mechanisms, including NET generation [29] to combat microorganisms. This happens more readily in the lungs compared with in other tissues, with neutrophils found to exist in higher concentrations in pulmonary vasculature compared with systemic blood vessels [30]. A prominent pathway leading to NET formation in infection is through the interaction of lipopolysaccharide (LPS), with toll-like receptor 4 (TLR4) found on neutrophils [31].

In patients with community-acquired pneumonia (CAP), increased levels of cell-free nucleosomes in serum samples, used as surrogate markers of NETosis, were found. This was associated with prolonged hospitalisation and a greater 30-day all-cause mortality [32]. This finding suggests NETs could function as a novel marker of prognosis in CAP.

#### Acute Lung Injury and Acute Respiratory Distress Syndrome

The degree of neutrophil influx into the lungs and NET release during ALI and ARDS positively correlates with disease progression and severity [17], with neutrophil depletion conferring protection in a transfusion-related ALI animal model [33]. NETosis seems to be a key component of ventilator-induced lung injury (VILI) [34] as evidenced by detection of citrullinated histone-3, suggesting that this was a mode of cell death independent from apoptosis and necrosis [35]. The authors suggested that this may be due to increased levels of IL-1β and HMGB1, which have been both shown to be able to induce NETosis.

#### Allergic Asthma

Patients with neutrophilic asthma have a greater severity of disease and reduced response to corticosteroid treatment compared with the eosinophilic type [34]. The increased expression of neutrophil chemoattractant IL-8 in airway smooth muscle cells could be contributing to disease severity [35] through inducing NETosis.

#### Chronic Obstructive Pulmonary Disease

NETosis has been documented as an integral part of chronic obstructive pulmonary disease (COPD) pathophysiology. Unlike asthma, where neutrophils are important in certain subtypes, NETosis has been directly linked to disease severity in COPD [36, 37]; TLR-4 expression, one of the main potentiators for NET formation, is increased during COPD exacerbations [38].

#### Pulmonary Hypertension

NETs are also able to potentiate dysregulated angiogenesis, as seen in patients with chronic thromboembolic pulmonary hypertension and idiopathic pulmonary hypertension as plasma levels of DNA, NE and MPO levels are significantly elevated. Moreover, NETs also seem to destabilise intercellular junctions and increase endothelial cell motility. Through direct contact with endothelial cells, NETs were found to induce the activity of the pro-inflammatory transcription factor NFκB by approximately 3-fold. Moreover, NETs increase the surface expression of von Willebrand Factor and platelet adhesion, thereby producing a prothrombotic state [39].

### Kidney

#### Glomerulonephritides

NETs have been visualised upon immunostaining renal biopsies from patients with SLE and antineutrophil cytoplasmic antibodies-associated vasculitis (AAV) [40] and may be at least partially responsible for activating complement pathways resulting in disease exacerbations [41]. These autoimmune conditions also seem to affect the patient’s ability to degrade NETs, amplifying their deleterious inflammatory effects [12]. In crescentic glomerulonephritis, neutrophil-mediated glomerular damage is worsened by addition of extracellular MPO, which could have been released during NETosis [42]. NETosis could also contribute to the loss of immune tolerance through further externalisation of crucial autoantigens during cell death [4].

#### Haemolytic Uraemic Syndrome (HUS)

Plasma from affected patients exhibited a greater capacity to undergo NETosis compared with healthy patients. The ensuing damage has been linked to the pro-inflammatory cytokines IL-6 and IL-8 released from glomerular epithelial cells, upon stimulation by NETs. This potentiates microvasculature inflammation and thrombosis, precipitating renal failure [43].

### Liver

#### Decompensated Cirrhotic Liver Disease

A deficiency in NET release has been demonstrated to play a role in the onset of end-stage liver disease [44] as neutrophils in cirrhotic patients are found to have defective ROS production [45], which commonly triggers NET release [46]. This may also partially explain why these have a predisposition to recurrent bacterial infections [47] and increased rates of decompensatory complications such as spontaneous bacterial peritonitis (SBP). This is corroborated by defective NET release from ascitic fluid neutrophils in cirrhotic patients compared with controls *in vitro* [48]. Cirrhotic patients with SBP were also found to have an increase in pro- and anti-inflammatory cytokines in peripheral blood and ascitic fluid [49].

#### Ischaemia-Reperfusion Injury

Ischaemia-reperfusion injury (IRI) is an inherent consequence of liver transplantation, hypovolaemia or trauma and results in the release of damage-associated molecular patterns (DAMPs) which, in turn, cause NET formation in a TLR-dependent manner, exacerbating inflammation [50]. Treatment with a peptidyl-arginine-deiminase 4 (PAD4) inhibitor [51] or DNase has been shown to be significantly hepatoprotective following liver IRI [50].

### Cardiovascular System

#### Atherosclerosis

NETs are a well-known constituent of atherosclerotic lesions [52]. MPO has been strongly associated with diminishing the cardioprotective effects of high-density lipoprotein cholesterol (HDL-C) through oxidation reactions, and an increased enzymatic activity is linked to increased plaque rupture [53]. Other proteins found in NETs, such as cathelin-related antimicrobial peptide (CRAMP), have also been shown to contribute to disease progression [54]. Moreover, *in vitro* studies show that hypercholesterolemia triggers NET formation [55]. A large-scale study in patients with suspected coronary artery disease revealed that the markers of NETosis, such as extracellular DNA, are independently associated with disease severity [56]. Coronary specimens from patients after an acute myocardial infarction (MI) showed the presence of NETs in both fresh and lytic thrombi, therefore suggesting NETosis happens in the early stages of thrombus evolution [57]. Furthermore, NET burden was shown to be positively correlated with the infarct size in patients undergoing primary percutaneous coronary interventions post-MI. This is supported by increased levels of MPO, DNA and NE in the lesion site [58]. Therefore, NETs could potentially be considered as a novel biomarker in atherosclerosis [59].

#### Diabetes Mellitus-Induced Vasculopathy

It has been shown that neutrophils form peripheral blood of diabetes mellitus (DM) patients showed increased spontaneous NETosis [60]. Interestingly, metformin reduces the deleterious effects of NETosis in a mechanism independently from glucose control. One recent study showed that 2-month treatment with metformin in pre-DM patients reduced levels of components of NETs, whereas glycaemic control with other medication such as insulin saw no difference when compared with placebo controls. This has been attributed to a direct effect of metformin on inhibiting the activation of NADPH oxidase [61].

### Musculoskeletal System

#### Rheumatoid Arthritis

Neutrophils from the peripheral blood and synovial fluid of patients with rheumatoid arthritis (RA) exhibit increased NETosis compared with healthy controls and patients with osteoarthritis. The externalisation of citrullinated proteins during the process of NETosis was found to initiate and perpetuate the aberrant immune response in RA. Moreover, the autoantibodies and inflammatory cytokines commonly seen in RA promote NETosis, resulting in a vicious cycle of tissue destruction [62].

#### Gout

Gout is an inflammatory disease that involves the deposition of monosodiumurate (MSU) crystals in joints. During acute gout, there is increased movement of neutrophils into the synovial fluid. MSU is a known neutrophil stimulus and it has been shown that acute gout is associated with an increase in IL-1β levels [63], a key player in NET formation.

### Gut

#### Ulcerative Colitis

There is prominent neutrophil infiltration in the colon mucosa in ulcerative colitis (UC) [64] and this correlates with disease severity. In UC, the inflammatory environment promotes neutrophil activation and IL-1β expression [65]. In contrast, NETs do not seem to play a key role in Crohn’s disease. This may explain why mesalazine, a known inhibitor of IL-1β production and the first-line treatment for UC flare-ups, is not therapeutic in Crohn’s patients *per se* [66].

### Reproductive System

#### Pre-eclampsia

Placentas from women diagnosed with pre-eclampsia showed increased neutrophil infiltration and NETosis when compared with non-hypertensive pregnant controls [67, 68] and are probably involved in causing widespread damage to the maternal endothelium [69].

#### Placental and Endothelial Injury During Pregnancy

Aberrant neutrophil activity during pregnancy is also associated with other severe complications, including recurrent foetal loss [70, 71]. One recent study indicated neutrophils in pregnant women seem to have an increased propensity to undergo NETosis, secondary to an increase in granulocyte colony-stimulating factor during pregnancy [72]. Progesterone has been shown to attenuate neutrophil-mediated ROS production, whereas 17β-estradiol induces intracellular ROS generation in a dose-dependent manner [73].

## NETosis AS A THERAPEUTIC TARGET

**Fig. 3:**
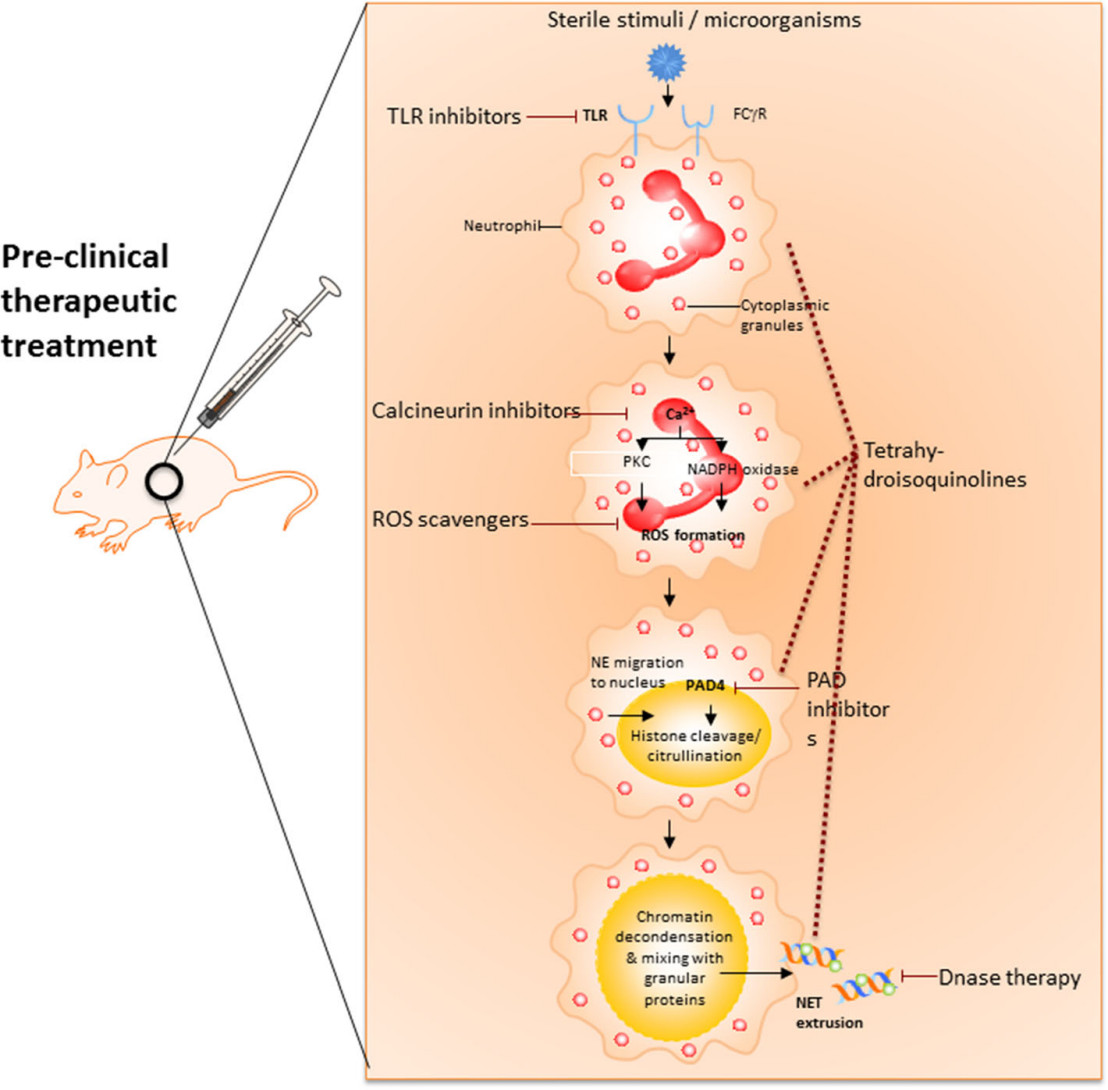
Therapeutic strategies targeting NET formation. Stimulation of neutrophil receptors (*e.g.* FC γ receptor, toll-like receptor) by microorganisms or sterile signals leads to release of calcium (Ca^2+^) from the endoplasmic reticulum (ER). Cellular Ca^2+^ overload results in activation of protein kinase C (PKC), assembly of the nicotinamide adenine dinucleotide phosphate (NADPH) oxidase complex, and/or mitochondrial activation, thus stimulating reactive oxygen species (ROS) production. Oxidative stress promotes myeloperoxidase (MPO)-dependent migration of granular neutrophil elastase (NE) into the nucleus to cleave histones. Subsequent activation of peptidylarginine deiminase (PAD) 4 induces histone citrullination to cause chromatin decondensation. The last step involves nuclear membrane degradation and extrusion of a mixture of chromatin and granular proteins into extracellular space, whereby extracellular DNase eventually digests and removes neutrophil extracellular traps (NETs). In this regard, modulation of critical steps in NET formation and degradation (shown by blocking arrows) might be beneficial for the treatment of inflammatory disorders (figure modified and reproduced with permission) (42). FcγR, Fc γ receptor; TLR, toll-like receptor.

Targeting critical steps in NET formation and degradation is critical for developing treatment strategies for NETosis-associated organ injury associated with NETosis (Fig. 3) [4]**.** Examples of recent publications on potential therapeutic compounds targeting NETosis are summarised in Table 1.

**Table 1:** Potential Therapeutic Approaches Targeting NETosis

Drug class	Study	Main findings
TLR inhibitors	Wan T *et al*. (2017)	Dexamethasone reduced NETosis in neutrophils stimulated with *S. aureus*. Agonists of TLR2 and TLR4 rescued dexamethasone inhibition. ROS production was unaffected by dexamethasone.
Calcineurin inhibitors	Gupta AK *et al*. (2014)	Cyclosporine A reduced the effect of key physiological stimuli that activate neutrophils, such as IL-8 and suppressed NETosis.
ROS scavengers	Patel *et al.* (2010) Vorobjeva NV and Pinegin BV (2016)	N-Acetyl cysteine reduced NET formation and severity of SLE in patients. Antioxidants Trolox and Tempol prevent NETosis of in stimulated human neutrophils in a dose-dependent manner.
PAD inhibitors	Knight JS *et al.* (2013) (2014)	11 weeks of daily Cl-amidine injections reduced NET-induced vascular damage and area of lesions in a murine model of atherosclerosis. PAD inhibition dampens disease activity by reducing endothelial dysfunction in a murine model of SLE.
DNase therapy	Papayannopoulos V, Staab D, Zychlinsky A. (2011)	Elastase combined with DNase therapy enhances solubilisation of sputum in cystic fibrosis patients.
Tetrahydroisoquinolines	Martinez NE *et al.* (2017)	Tetrahydroisoquinolines selectively target NET overproduction at micromolar concentrations, possibly at multiple stages of NET formation, without compromising normal neutrophil function.

### TLR Inhibitors

Dexamethasone (Dex) has been shown *in vitro* to reduce NETosis in neutrophils that are stimulated with *Staphylococcus aureus* but not in those stimulated with PMA. The TLRs involved in *S. aureus*-induced NET formation seem to be TLR2 and TLR4, as agonists of these receptors rescued Dex inhibition. Interestingly, although Dex reduced NET formation, it did not significantly affect ROS production [74].

### Calcineurin Inhibitors

Calcineurin is a calcium-dependent serine/threonine protein phosphatase that is important for neutrophil activity. Many stages of NETosis induction depend upon calcium mobilisation. Hence, modulators of the calcineurin pathway are potential pharmacological inhibitors of NET formation. Cyclosporine A (CsA), an antagonist of the calcineurin pathway, has been shown to reduce the effect of key physiological activators of neutrophils. This effect on NETosis may in part explain CsA’s efficacy in RA [75] and steroid-resistant UC patients [76]. PMA-induced NETosis seems to be calcium-independent, as this was not inhibited by CsA [77].

### ROS Scavengers

The mitochondria are a powerful source of ROS [78]. ROS scavengers such as N-acetyl cysteine (NAC) reduce NET formation and severity of SLE in patients [79]. Trolox and Tempol are two antioxidants which have also been shown to prevent NETosis of PMA-stimulated human neutrophils in a dose-dependent manner and have been recommended for treatment of autoimmune and inflammatory pathologies [80].

### PAD Inhibitors

Using a murine model of atherosclerosis, Knight and colleagues have shown that pharmacological inhibition of PAD4 using 11 weeks of daily Cl-amidine injections reduced NET-induced vascular damage, with delayed plaque progression in the carotid arteries [81]. The same group also showed that PAD inhibitors reduce disease activity in murine models of SLE, by reducing endothelial dysfunction [82]. It is worth mentioning that the possibility of PAD inhibition as a therapeutic avenue to be pursued in NET-induced organ damage in glomerulonephritis has been recently challenged by the work of Gordon and colleagues on murine models on SLE with PAD4 deletion. They showed that this did not reduce end-organ damage as measured by proteinuria [83], suggesting that mechanisms other than NET formation are implicated in this complex autoimmune condition.

### DNase Therapy

DNase therapy has been proposed to improve outcomes in CF patients through reducing mucous viscosity. However, it appears that recombinant DNAse does not reduce the load of DNA/protein complexes seen in NETosis. One solution to this is to combine elastase with DNAse, in order to degrade histones and provide DNAse access to chromatin. This combination has been shown to enhance solubilisation of sputum [84].

### Tetrahydroisoquinolines

In contrary to the aforementioned mechanisms of NETosis inhibitors, tetrahydroisoquinolines (THIQs) are a new class of NET formation inhibitors that do not target ROS formation or granular protein activity. As functional neutrophils are paramount to maintaining immune reactivity, this difference offers an advantage to selectively target NET overproduction without impairing normal function. The exact molecular mechanisms of THIQs are yet to be determined; however, it is known that THIQ inhibition of NETosis take place at micromolar concentrations and possibly at different stages of NET formation [85].

## CONCLUSION

When regulated as part of normal physiology, NETs are anti-microbial and fundamental to the innate immune system. Dysregulated NET formation contributes to the pathogenesis of a plethora of diseases. This review has summarised the role of NETosis in pathologies of multiple body systems, as well as highlighted the stages of NETosis that has so far been investigated as emerging pharmacological targets. These putative strategies seem to hold therapeutic potential and warrant further investigation.

## Abbreviations

NET: neutrophil extracellular traps
PMN: polymorphonuclear
PAD4: peptidyl arginine deiminase 4
NADPH: nicotinamide adenine dinucleotide phosphate
ER: endoplasmic reticulum
PKC: protein kinase C
ROS: reactive oxygen species
NE: neutrophil elastase
MPO: myeloperoxidase
mtDNA: mitochondrial DNA
SLE: systemic lupus erythematosus
PMA: phorbol 12-myristate 13-acetate
CDK: cyclin-dependent kinases
AD: Alzheimer’s disease
*CF*: cystic fibrosis
SP-D: surfactant protein D
LPS: lipopolysaccharide
TLR4: toll-like receptor 4
CAP: community-acquired pneumonia
ALI: acute lung injury
ARDS: acute respiratory distress syndrome
VILI: ventilator-induced lung injury
HMGBI: High Mobility Group Box 1
COPD: chronic obstructive pulmonary disease
AAV: antineutrophil cytoplasmic antibodies-associated vasculitis
HUS: haemolytic uraemic syndrome
SBP: spontaneous bacterial peritonitis
IRI: ischaemia-reperfusion injury
DAMP: damage-associated molecular patterns
PAD4: peptidyl-arginine-deiminase 4
HDL-C: high density lipoprotein cholesterol
CRAMP: cathelin-related antimicrobial peptide
MI: myocardial infarction
DM: diabetes mellitus
RA: rheumatoid arthritis
MSU: monosodiumurate
UC: ulcerative colitis
CsA: cyclosporine
NAC: N-acetyl cysteine
THIQ: tetrahydroisoquinolines
